# Stem Cell Therapies for Treatment of Liver Disease

**DOI:** 10.3390/biomedicines4010002

**Published:** 2016-01-06

**Authors:** Clara Nicolas, Yujia Wang, Jennifer Luebke-Wheeler, Scott L. Nyberg

**Affiliations:** 1 William J. von Liebig Center for Transplantation and Clinical Regeneration, Mayo Clinic, Rochester, MN 55905, USA; wang.yujia@mayo.edu (Y.W.); luebke-wheeler.jennifer@mayo.edu (J.L.-W.); nyberg.scott@mayo.edu (S.L.N.); 2 Department of Surgery, Mayo Clinic, Rochester, MN 55905, USA

**Keywords:** stem cell, liver disease, induced pluripotent stem cells, gene correction, cell transplant, bioartificial liver, regenerative medicine, cell therapy

## Abstract

Cell therapy is an emerging form of treatment for several liver diseases, but is limited by the availability of donor livers. Stem cells hold promise as an alternative to the use of primary hepatocytes. We performed an exhaustive review of the literature, with a focus on the latest studies involving the use of stem cells for the treatment of liver disease. Stem cells can be harvested from a number of sources, or can be generated from somatic cells to create induced pluripotent stem cells (iPSCs). Different cell lines have been used experimentally to support liver function and treat inherited metabolic disorders, acute liver failure, cirrhosis, liver cancer, and small-for-size liver transplantations. Cell-based therapeutics may involve gene therapy, cell transplantation, bioartificial liver devices, or bioengineered organs. Research in this field is still very active. Stem cell therapy may, in the future, be used as a bridge to either liver transplantation or endogenous liver regeneration, but efficient differentiation and production protocols must be developed and safety must be demonstrated before it can be applied to clinical practice.

## 1. Introduction

As of today, liver transplantation is the only proven treatment for a wide variety of liver diseases refractory to medical treatment. Unfortunately, the demand for donor organs considerably exceeds their supply, making therapeutic alternatives to whole-organ liver transplantation necessary. Cell therapies have emerged in response to the shortage of transplantable livers; both *ex vivo* liver support therapy and *in vivo* cell transplantation have been evaluated and shown potential for the treatment of liver failure. The liver is particularly amenable to this form of therapy due to its high capacity for endogenous regeneration and repair [1,2,3].

Isolated primary hepatocytes were the first type of cell to be tested in both *in vivo* and *ex vivo* cell therapies, but their use has been limited by a number of technical difficulties that have yet to be overcome. Hepatocytes do not survive long in *in vitro* culture [4] because (1) *in vitro* growth capacity is minimal [5], (2) expression of liver-specific genes declines rapidly *in vitro* [6], and (3) susceptibility to freeze-thaw damage makes cryopreservation complicated [7]. The main limitation for their use, however, is that clinical demand for hepatocytes cannot be met due to a scarcity of donor livers from which high-quality primary hepatocytes can be isolated.

With the advent of regenerative medicine, the focus of liver cell therapy has shifted slightly onto the therapeutic potential of stem cells as a means to restore normal structure and function after tissue injury. The capacity of stem cells for differentiation and self-renewal make them a plausible source for the generation of unlimited numbers of hepatocytes. Therefore, stem cell therapies as an alternative for whole-organ liver transplantation hold great promise for the treatment of liver disease. Several types of stem cells have been proven to be appropriate for liver cell replacement. In this review, we address the advantages and limitations of each cell line, as well as the different liver diseases that may be able to benefit from stem cell therapy.

## 2. Stem Cell Sources for Liver Disease Therapy

### 2.1. Liver-Derived Stem Cells

Stem cells can be obtained from either adult or fetal livers. Both adult liver stem cells, also known as oval cells, and fetal liver stem cells, termed hepatoblasts, are bipotent and therefore able to differentiate into hepatocytes or bile duct cells [8,9,10]. Oval cells have been proven to play a part in liver regeneration when the replication capacity of hepatocytes is impaired [11], while hepatoblasts have been used experimentally to repopulate the liver in animal models [12,13]. Human hepatoblasts have also been cultured, and have shown *in vivo* engraftment and differentiation after transplantation into immunodeficient mice [14].

The major limitation to the use of liver derived stem cells is that their number within a normal liver is very low, with oval cells comprising only 0.3% to 0.7% of the adult liver [15], and hepatoblasts comprising less than 0.1% of the fetal liver mass [16]. This makes their isolation and expansion challenging, restricting their application to small-scale use.

### 2.2. Bone Marrow-Derived Stem Cells

Bone marrow-derived stem cells include hematopoietic and mesenchymal stem cells (MSCs) [17]. MSCs are multipotent progenitor cells found in bone marrow and other adult organs and tissues, such as adipose tissue, that are easily accessible and can be expanded rapidly in culture [18,19].

Out of these two cell populations, MSCs have been suggested to have a higher potential for liver regeneration [20]. In addition, they offer another advantage over hematopoietic stem cells: they have immunomodulatory or immunosuppressive properties that downregulate T cell, B cell, and NK cell function [21]. Clinically, this can translate into the ability to induce tolerance after liver transplantation.

### 2.3. Annex Stem Cells

Annex stem cells are easily accessible cells derived from human placental tissue, umbilical cord and cord blood, and amniotic fluid. They are pluripotent, so they have a higher differentiation potential when compared to adult stem cells, as well as a higher proliferation rate [22,23,24]. Annex stem cells also offer another advantage: they have not been described to form teratomas or teratocarcinomas in humans. In one study, intraperitoneal administration of human umbilical cord stem cells into non-obese diabetic severe combined immunodeficient mice after acute toxic liver injury demonstrated rapid liver engraftment, differentiation into hepatocytes, improved liver regeneration, and reduced mortality rates [25].

### 2.4. Embryonic Stem Cells (ESCs)

ESCs are totipotent cells that can be differentiated into hepatocyte-like cells with the ability to colonize the liver after injury and function similarly to mature hepatocytes [26]. There are two main limitations to the use of ESCs, however. In the first place, the fact that their procurement involves the destruction of embryos raises ethical concerns that have curbed the progress of ESC research [27]. Secondly, the issue exists of immune incompatibility between donors and recipients of ESC transplants [28]. Nevertheless, ESC research is still ongoing, and a recent study revealed an efficient protocol for differentiation into neonatal hepatocytes that were able to repopulate livers *in vivo* without tumor induction and rescue liver function in mice with acetaminophen-induced toxicity [29].

### 2.5. Induced Pluripotent Stem Cells (iPSCs)

**Figure 1:**
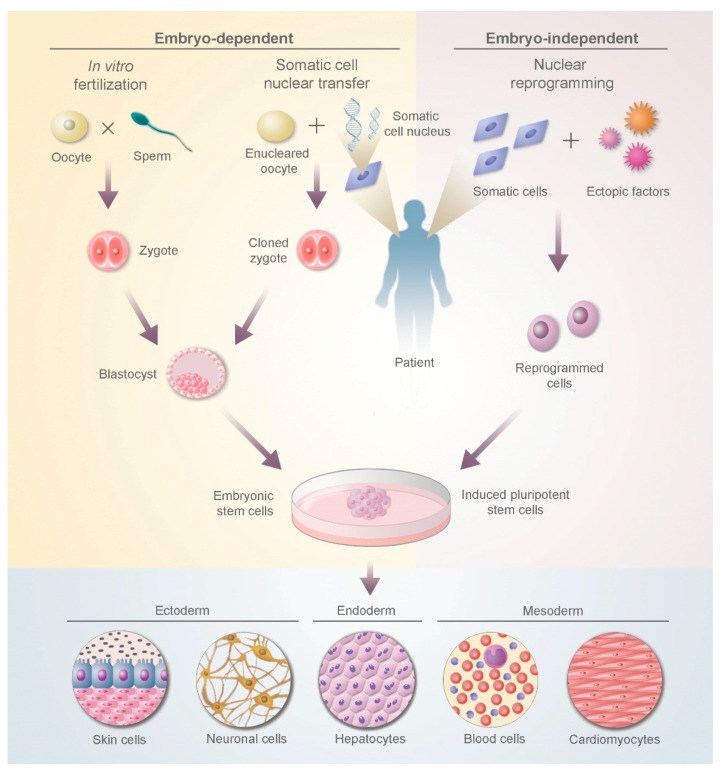
Production of embryonic stem cells and induced pluripotent stem cells.

iPSCs have similar properties to ESCs, including pluripotency and self-renewal, but sidestep the major concerns inherent to the use of this type of cells. iPSCs are produced *in vitro* from somatic cells, bypassing the use of embryonic tissue or oocytes and thereby avoiding ethical controversy [30]. Furthermore, they offer the possibility for autologous use, solving the problem of allogeneic rejection. The use of iPSCs, which were first described in 2006 [31], has rapidly grown as a promising alternative to embryonic stem cells, but before their clinical application may be considered several questions must be addressed, and their equivalence to ESCs must be ascertained [32]. Figure 1 shows the differences in production between iPSCs and ESCs.

iPSCs may be generated from diverse cell sources, and it has been suggested that their origin may play a role in their capacity to differentiate [33,34]. Although fibroblasts are the most common source of human iPSCs (hiPSCs), these cells have also been reprogrammed successfully from a variety of other somatic cell types, including primary hepatocytes [35]. It has been suggested, however, that hepatocyte-derived cell lines may have a higher propensity towards teratoma formation than cells from other origins [36]. Evidence exists that hepatic differentiation may be induced successfully from iPSCs of diverse origins [37], because the hiPSC line’s epigenetic memory is lost over time [38]. In fact, studies have shown that predictable iPSC differentiation is independent of somatic origin, but rather is greatly dependent on the reprogramming strategy used [39]. Other authors have suggested that on the contrary iPSCs have a skewed differentiation potential that stems from their lineage-specific epigenetic memory, predisposing them to differentiate more readily into their cell type of origin [40]. Further research is needed to clarify this point and find the most adequate cell source for generating hiPSCs to differentiate into hepatocytes. Different methods of reprogramming iPSCs also exist, with the first reports of iPSC generation consisting of retroviral vectors being used to induce pluripotency. This method is limited by the possibility of spontaneous reactivation of the viral transgenes and their integration into the host genome, which translates into a risk of tumor formation [41]. However, the issue of iPSC generation has been addressed successfully over the past two years. For example, hiPSCs can now be generated by vectors which do not integrate into the target cell’s genome [42,43], and can even be induced by small molecule compounds [44,45].

To date, fully mature hepatocytes have not been obtained *in vitro* from iPSCs nor from ESCs. The cells obtained, termed hepatocyte-like cells (HLCs), share most of the properties of primary hepatocytes but are not as functionally mature, as is demonstrated by their lower levels of albumin production, CYP activity, and urea cycle activity, as well as by their persistent expression of high levels of α-fetoprotein [46]. Numerous differentiation protocols have been developed [47,48,49], and their efficiency has been increased through a reduction in differentiation time from an average of over 20 days to 12 days with a three-step protocol [50,51]. Asgari *et al.* [52] characterized hiPSC-derived HLCs as expressing hepatocyte-specific markers, glycogen and lipid storage activity, albumin secretion, and CYP450 metabolic activity, and after transplantation these cells had the ability to improve the functional status of a CCl4-injured mouse liver [52]. The latest studies, which focus on direct lineage reprogramming of fibroblasts into human induced hepatocytes have yielded functional and expandable cells with drug metabolic function [53,54]. Nonwithstanding, differentiation protocols must be optimized before clinical application is feasible, as it has been suggested that fully differentiated cells bear lower risks of teratoma formation after transplantation [55]. Residual undifferentiated cells must be eliminated to avoid not only teratoma formation, but also the possibility of an immune response to pluripotency antigens [56]. Furthermore, the immunologic properties of the cell type of interest must be carefully studied after differentiation [57,58].

Another obstacle that must be overcome is the lack of an efficient, large-scale production system for hiPSCs, as monolayer static tissue cultures will not be able to sustain the rapid cell expansion necessary for clinical application. Advances have been made on this front, with iPSC culture as aggregates in 3D suspension [59]. 3D culture offers the advantage of culturing hiPSCs at high densities [60], and at the same time increases functional maturation of HLCs towards an adult phenotype and improves their functional longevity [61].

Despite these setbacks, promising results have been attained. Zhu *et al.* [62] were able to differentiate hepatocytes from human fibroblasts through induced multipotent progenitor cells (iMPCs) instead of iPSCs by shortening the reprogramming protocol to avoid pluripotency [62]. They then achieved liver repopulation in an immunodeficient mouse model of human liver failure, with hepatocyte function levels similar to those of human adult primary hepatocytes. Furthermore, by blocking cells from entering a pluripotent state, tumor formation was likely prevented. Both tumorigenicity and immunogenicity of iPSCs have been found to decrease with reprogramming methods that do not involve genomic integration [63], as well as by removing c-Myc during reprogramming [64]. With regard to the tumorigenic potential of iPSCs, an autologous teratoma formation assay was recently performed in the rhesus macaque, and it concluded that while undifferentiated autologous iPSCs form teratomas, iPSC-derived progenitor cells give rise to functional tissues *in vivo* with no signs of tumor formation [65]. Furthermore, the teratomas that were formed by undifferentiated cells were at least twenty times less efficient in their growth than in an equivalent rodent model, probably due to the presence of an intact immune and inflammatory system. Its similarities to human physiology make this nonhuman primate model very valuable for the study of iPSC-based therapies.

iPSCs have been studied in the context of a number of different liver diseases, but their most immediate use is probably the modelling of human liver disease and testing of drugs *in vitro* [66,67].

## 3. Techniques in Stem Cell Use for Liver Disease Therapy

**Figure 2:**
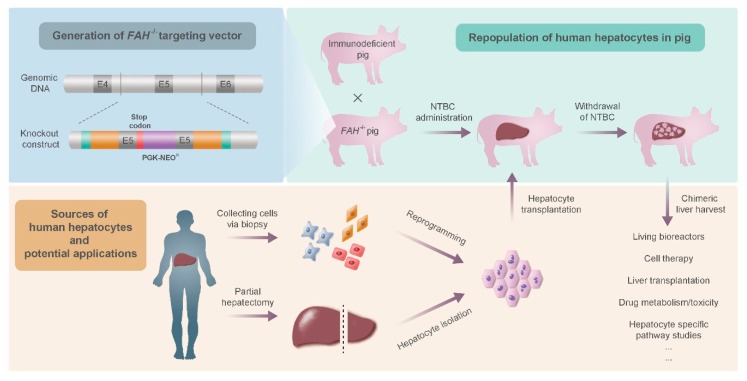
Repopulation of FAH-deficient pig livers with human hepatocytes. Nitisinone (NTBC) is used to treat FAH-deficient animals while hepatocyte engraftment and proliferation takes place.

Differentiation of stem cells into HLCs can be achieved *in vitro* prior to their use or *in vivo* after cell transplantation. *In vitro* culturing and differentiation is being studied extensively, and new, more efficient protocols are still being created. However, stem cells also have the ability to differentiate into HLCs *in vivo* after injection. This is true of different cell lines. In one study, intravenous injection of purified hematopoietic stem cells showed *in vivo* differentiation to HLCs in a fumarylacetoacetate hydrolase (FAH) knockout mouse, restoring liver function [68]. FAH^−/−^ mice are animal models of tyrosinemia type I, and possess great potential for the study of regenerative treatments for metabolic liver disease. In these animals, non-FAH deficient wild-type cells can extensively repopulate the liver upon transplantation due to their selective advantage. When the FAH knockout is combined with immunodeficiency alleles, human cells can be used to repopulate the liver, creating a chimeric organ [69]. To provide a more clinically relevant animal model, FAH^−/−^ pigs have also been created that can be used to test the efficacy of different cell therapy approaches [70]. Furthermore, if liver humanization can be adequately achieved in these animals, they could be exploited as living bioreactors for the production of large quantities of functional human hepatocytes. Figure 2 shows the creation of FAH knockout pigs and their possible use as incubators for primary human hepatocytes.

Other cell lines, such as mouse ESCs and human bone marrow-derived MSCs, have also been proven to differentiate into HLCs *in vivo* [71,72]. Notwithstanding, both *in vitro* and *in vivo* signal patterns, mechanisms of differentiation, and optimum proliferation conditions must be studied in more depth before clinical applications can be considered, especially since the data suggest that engraftment and repopulation capacity is higher in mature hepatocytes than stem cells [73].

## 4. Potential Applications of Stem Cells in Liver Diseases

### 4.1. Hereditary Liver Diseases

Cell therapy in hereditary liver diseases can not only serve as a bridge to liver transplantation, but also offers the opportunity for long-term correction of the metabolic deficiency [74]. Primary hepatocyte transplantation has been used for treatment of several diseases in humans, including familial hypercholesterolemia, Crigler-Najjar syndrome type 1, and urea cycle defects among others [75]. Patients with both Crigler-Najjar type 1 and urea cycle defects are undergoing a phase I trial for treatment with a heterologous human adult liver progenitor cell suspension generated from normal adult liver tissue [76]. As discussed earlier, however, there is a shortage of donor organs from which to isolate high-quality hepatocytes, and the possibility of allogeneic rejection must be taken into account. Allogeneic rejection may be avoided with autologous transplantation, but autologous primary hepatocytes can only be obtained in sufficient number through liver resection. This issue can be circumvented through the use of iPSCs.

**Figure 3:**
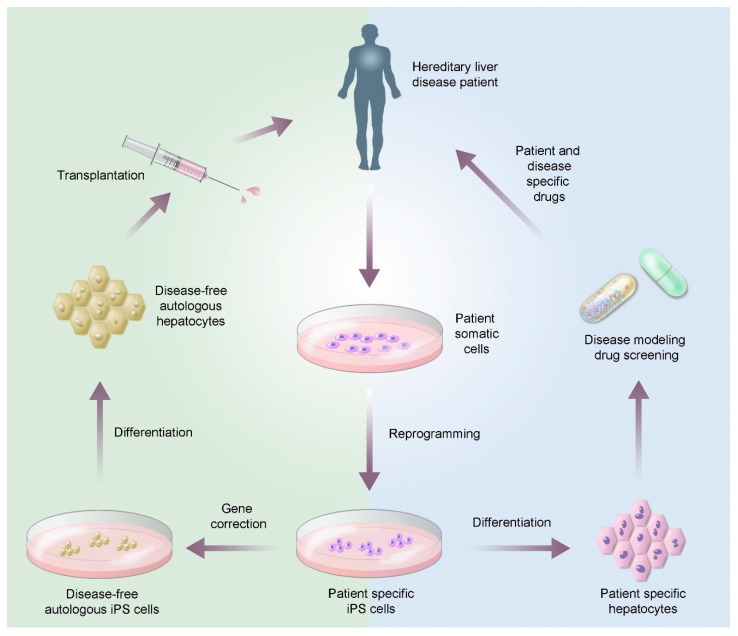
Gene correction of iPSC for the production of patient-specific disease-free hepatocytes.

With the development of stem cell technology, and particularly the development of iPSCs, hereditary liver disease treatment can be taken a step further: by combining genetic correction technology with autologous cell transplantation, patient-specific therapies can be created [77,78]. Disease-free autologous hiPSCs are first generated through *ex vivo* gene therapy [79], and the genetically-corrected hiPSCs then differentiated and used for transplantation. Figure 3 shows how hiPSCs can be combined with gene correction and differentiation techniques to produce autologous, disease-free hepatocytes for transplantation.

In theory, this method could be applied to any hereditary disease for which the underlying mutation is known, and it has been tested on animal models of hematopoietic diseases with encouraging results [80]. α1-antitrypsin deficiency has also been genetically corrected in hiPSCs, restoring the protein’s function in the subsequently differentiated HLCs both *in vitro* and *in vivo* in mice [81]. Disease-corrected HLCs have also been successfully produced from a familial hypercholesterolemia patient’s hiPSCs [82]. In light of these studies, transplantation of hepatocytes derived from autologous genetically corrected hiPSCs shows promise for the treatment of hereditary liver diseases. A patient-specific, disease-free line of hiPSCs can be obtained in 4–5 months [83]. Alternatively, hiPSC can be useful in the modeling and study of inherited metabolic disorders [51].

### 4.2. Acute Liver Failure (ALF)

As mentioned earlier, the liver has a considerable capacity for endogenous regeneration [84]. When it suffers an acute injury, repair mechanisms are put into effect that in many cases would be able with time to recover a functioning, viable liver [85], but liver function must be supported while regeneration takes place [86]. This has been attempted in two different ways: through cell transplantation or through a bioartificial liver (BAL) system.

Cell transplantation can offer a temporary solution to ALF or acute-on-chronic liver failure. Pareja *et al.* [87] performed hepatocyte transplantation in acute-on-chronic liver failure patients with encouraging results, including improvement of hyperammonemia and degree of encephalopathy [87]. Similarly, transplantation of immortalized human fetal hepatocytes significantly improved survival in mice after 90% hepatectomy [88]. Coencapsulation of hepatocytes with bone marrow-derived MSCs not only augments engraftment [89] and lengthens hepatocyte viability, but also enhances hepatocyte-specific functions both *in vitro* and *in vivo* [90]. When transplanted on their own, bone marrow-derived MSCs both ameliorated liver damage and suppressed intrahepatic NK cell activity in mice [91,92]. Furthermore, there is evidence that the immunomodulation produced by an MSC-conditioned medium alone would be sufficient to potentially eliminate the need for donor hepatocytes [93]. MSC-derived exosomes have also been shown to activate a regenerative response resulting in a higher expression of proliferation proteins in CCl4-injured mice [94]. The advantage that these treatment options offer over a cell-based therapy is that they are less likely to trigger an immune response. With regard to iPSC, Chen *et al.* [50] demonstrated that after application of their three-step differentiation protocol, iPSC-derived HLCs rescued lethal fulminant hepatic failure in a severe combined immunodeficient mouse model [50].

Another promising treatment for ALF is the BAL, a form of extracorporeal supportive therapy that removes toxins while performing the biotransformation and synthetic functions of active hepatocytes [95]. This system is intended to bridge patients with ALF to either recovery of the native liver through regeneration or to liver transplantation [96]. The first BAL to be approved for a phase II/III trial was a porcine hepatocyte-based device, which was evaluated in a prospective, randomized, controlled trial in patients with ALF. Survival was improved in a subgroup analysis of patients with fulminant or subfulminant hepatic failure, but the primary endpoint of survival for the entire study population was not met [97].

Although primary porcine hepatocytes are the most commonly used cell source for BAL trials [98], immortalized C3A human hepatoblastoma cells have also been tested in the Extracorporeal Liver Assist Device (ELAD) [99], although no randomized controlled trials have shown survival benefit to date and meta-analysis results are inconclusive [100,101]. HepaRG cells, a human hepatic bipotent progenitor cell line [102] able to differentiate into hepatocyte clusters and surrounding biliary epithelial-like cells after exposure to dimethyl sulfoxide (DMSO) [103], are now being evaluated for BAL application in the Amsterdam Medical Center (AMC) bioreactor, with mixed results [104,105]. The HepaRG-AMC-BAL has been shown to increase survival time in ALF rats compared to acellular BAL treatment [106].

In order for the clinical application of the BAL to be successful, it appears that at least 200 g of functional hepatocytes must be accessible for each treatment. For this reason, the use of primary human hepatocytes is currently not practical due to both their limited availability, and their short functionality and viability *in vitro*. These issues have been addressed with the use of hepatocyte spheroids, which protect the cells from apoptosis and allow a greater cell mass to be used during treatment [107]. The use of porcine hepatocytes has also been limited by concerns of xenogenicity and xenozoonosis, while the use of immortalized cell lines has been limited by their loss of essential cell functions, such as urea cycle and CYP enzyme activity [108]. Consequently, ESC and iPSC are promising cell sources for BAL devices. Soto-Gutierrez *et al.* [109] showed that treatment of ALF with a subcutaneously implanted BAL seeded with ESC-derived HLCs in 90% hepatectomized mice improved their liver function and prolonged their survival [109]. A preliminary study with iPSC also exists that indicates these cells were differentiated into HLCs after a seven-day culture in a bioreactor module [110].

One of the major limitations to the use of stem cells for the treatment of ALF is the time constraint. ALF therapy requires a rapid and efficient production of large quantities of cells so that the time needed to culture and differentiate autologous cells, as per the current protocols, may be prohibitive, making allogeneic hepatocytes a more practical option. The use of iPSC banks with close HLA/MHC match to the ALF patient, once effective and rapid differentiation protocols into HLCs have been established, is an option that requires further study [108].

### 4.3. Cirrhosis

The treatment of chronic liver disease and cirrhosis focuses on repairing the disruption of the original liver structure, as well as on improving homeostasis and liver function. While HLCs may be useful for the support of impaired liver function, they do not seem to have a significant effect on curbing collagen deposition and restoring normal tissue architecture. It is mesenchymal bone marrow stem cells that have shown the most benefit in this area.

Small phase I trials have reported modest improvement in albumin levels, Child-Pugh and MELD scores after autologous bone marrow cell infusion [111,112]. When compared to granulocyte-colony-stimulating factor mobilization therapy alone, peripheral blood monocyte transplant therapy offered a significant improvement in liver function as measured by albumin levels and Child-Pugh scores [113]. Another study found that after intraparenchymal autologous mesenchymal bone marrow-derived stem cell transplantation in hepatitis C patients, the liver showed structural improvement as evidenced by its decreased extracellular matrix protein count on biopsy [114]. Hepatitis C patients have also been treated with peripheral injection of autologous MSCs, with MELD score improvement but no change in liver fibrosis or regeneration on histopathological examination at six months post-treatment. HCV RNA levels became negative in patients with non-responder hepatitis C, suggesting that these cells have an immunomodulatory effect [115]. In a liver fibrosis rat model, adipose tissue-derived MSC infusion suppressed fibrosis progression and slightly ameliorated liver function. This therapeutic effect was enhanced by basic fibroblast growth factor treatment, possibly due to a higher hepatocyte growth factor expression [116]. It has been proposed that adipose tissue-derived MSCs may be superior to other cell lines due to their easy accessibility and immune privilege: they suppress activated lymphocyte proliferation and inhibit inflammatory cytokine production. They are also capable of differentiating into HLCs *in vivo* and reducing liver fibrosis by secreting metalloproteinase; a phase I/IIa trial will begin shortly [117]. Another study showed that infusion of human umbilical cord-derived MSCs improved insulin resistance in rats after CCl4-induced liver cirrhosis [118]. Stem cells from human exfoliated deciduous teeth have also been used in CCl4-treated mice with successful engraftment and *in vivo* differentiation to HLCs, as well as a decrease in the total area of liver fibrosis. Secondary transplantation of these HLCs into another CCl4-injured mouse improved liver function in these animals [26].

Other cell lines have also been postulated as candidates for the treatment of liver cirrhosis over the past year. This includes transplantation of hepatic stem or progenitor cells and transplantation of human fetal biliary tree stem or progenitor cells. Hepatic stem/progenitor cells demonstrated the ability to engraft, proliferate, differentiate into HLCs, and repopulate a thioacetamide-induced fibrotic rat liver. Both active fibrogenesis and net fibrosis were reduced [119]. Human fetal biliary tree stem/progenitor cells were transplanted via hepatic artery infusion in patients with advanced cirrhosis, resulting in clinical and biochemical improvement that remained stable for 6–12 months [120].

In the same way as MSCs appear to be optimum candidates for the reversal of fibrosis and inflammatory changes, ESCs or iPSCs may be excellent candidates for liver function support through the production of HLCs. Therefore, co-transplantation HLCs and MSCs together may offer a significant benefit in terms of both restoring liver function and alleviating the inflammatory microenvironment [121]. It has also been hypothesized that the paracrine effects of MSCs may go beyond enhancement of liver regeneration, and aid in HLC engraftment [122]. Furthermore, Espejel *et al.* [123] showed that not only do iPSC-derived HLCs provide normal liver function in mice, but they also replicate the unique proliferative capabilities of wild-type hepatocytes [123].

### 4.4. Liver Cancer

Stem cell therapy in liver cancer has two potential benefits. The first is to increase liver function after embolization or resection; the second is to take advantage of the graft-versus-tumor effect that occurs in allogeneic transplants. The liver has a great capacity for regeneration after partial hepatectomy. However, when the functional liver remnant (FLR) volume reaches a critical limit of 25% the risk of postoperative liver failure increases significantly [124]. In these cases, intraportal administration of autologous CD133(+) bone marrow-derived stem cells after portal venous embolization of several liver segments has been shown to increase proliferation rates and FLR volume [125,126]. Similarly, stem cell therapy prior to surgical liver resection may result in significant improvement in liver function parameters, as well as a decrease in post-operative complications [127]. Potentially, cell therapy could be used to replace or repair liver tissue damaged not only by surgery, but also by radiation or chemotherapy.

At the same time, adjuvant cell therapy with donor lymphocytes has been linked to more favorable tumor response rates in solid tumors, and particularly in patients who developed graft-versus-host disease, suggesting that allogeneic cell transplantation of hematopoietic stem cells augments the graft-versus-tumor effect of chronic graft-versus-host disease [128]. Adoptive immunotherapy, based on the transfer of naturally occurring or genetically engineered T cells, is another novel form of cancer therapy that has been proven to lower recurrence rates when applied postoperatively to the treatment of hepatocellular carcinoma [129,130]. This same technique could be performed using iPSCs, which are able to provide an unlimited source of highly reactive antigen-specific cytotoxic T-lymphocytes that, when transferred into the patient, can target, infiltrate, and eradicate tumors [131]. Stem cells, in this case bone marrow-derived MSCs transduced with a lentiviral vector stTRAIL (trimeric human necrosis factor-related apoptosis-inducing ligand in its secretable form), have also been used to treat heat-shocked residual cancer cells after radio frequency ablation in rats, resulting in tumor growth inhibition and significantly increased survival [132].

The most immediate use of stem cells, however, may be *in vitro* screening of new antitumor drugs, as they are able to provide cellular targets containing all of the patient’s tumor’s mutations [133]. This is true also for predicting individual differences in both drug metabolism and response, which can be recreated through hiPSC-derived HLCs [134].

### 4.5. Liver Transplantation

Liver transplantation is the only proven treatment for most end-stage liver diseases, but its use is limited by the insufficient number of donor organs. In response to the shortage of deceased donor livers available for transplantation, split liver and living donor liver transplantation were developed [135]. These techniques utilize small-for-size livers, with the size of the graft being directly related to the degree of liver function [136], so that they depend on rapid regeneration of the size-mismatched graft to support an adequate liver function [137]. This is especially important because liver regeneration, although augmented in half-size grafts, is suppressed in quarter-size grafts [138] and suppression of graft regeneration has been postulated to be the primary cause of small-for-size syndrome after partial liver transplantation in mice [139]. Consequently, therapeutic efforts must focus on both enhancing graft regeneration and supporting liver function throughout the early post-transplant period. In rats, MSCs genetically modified to over-express hepatocyte growth factor decreased hepatocellular injury marker levels, promoted liver weight recovery and preserved liver architecture after small-for-size transplants [140,141]. Aside from improving liver regeneration, MSCs also appear to prolong survival in these animal models of small-for-size liver transplant [142]. In reduced-size rat liver transplantation, MSC-conditioned medium infusion provided significant survival benefit by preventing release of liver injury biomarkers, inhibiting sinusoidal endothelial cell and hepatocellular death, and stimulating cellular proliferation [143]. Due to the importance of T-cell recruitment during ischemia-reperfusion injury, the immunomodulatory properties of MSCs and their potential for T-cell inhibition may be particularly useful in liver transplantation [144].

In the future, bioengineered organs could also be a solution to the shortage of donor livers [145], and stem cells are currently being studied as an alternative source of cells to hepatocytes [146]. Natural extracellular matrix components have been successfully utilized to induce embryonic stem cell differentiation into HLCs [147]. iPSCs show promise in this department, but a better understanding of stem cells and the processes involved in their differentiation is necessary before a consensus can be reached as to which cell line would be a safe and more efficient candidate for whole-organ bioengineering [148]. Another approach to organ creation is the production of liver buds *in vitro* from hiPSCs. This feat was performed successfully by Takebe *et al.* [149], who then proceeded to mesenteric transplantation of these liver buds in immunodeficient mice with ganciclovir-induced liver failure. Not only did the liver buds demonstrate protein production and human-specific drug metabolism, but their transplantation rescued the drug-induced lethal liver failure model [149]. Although orthotopic liver transplantation is not possible with this technique, and as of yet these liver models lack external biliary structures, this accomplishment is a first step towards generating new organs for transplantation [150].

## 5. Conclusions

Stem cell therapies and regenerative medicine may in the future provide solutions to the shortage of livers available for transplantation. Different lines of cells have been tested as potential therapy sources for a variety of liver diseases. Cell transplantation, together with organ engineering techniques and the BAL system, may offer an alternative to liver transplantation in these patients or at least decrease waitlist mortality rates [151]. Furthermore, with the development of iPSCs in the last decade ethical and immune issues have been partially circumvented, possibly accelerating research in this field.

Before many of these cell therapies are ready for clinical application, however, a number of important issues must be addressed. Firstly, efficient differentiation into mature hepatocytes must be achieved without the use of viral vectors or changes in cell cycle regulators so as to avoid tumorigenicity concerns and provide a better understanding of the cellular signaling process involved [152,153]. This must be accompanied by a reliable method for rapid, large-scale production of high-quality cells for transplantation. Finally, prior to clinical application these techniques should be tested and proven successful in large animal models, as they are better predictors of responses in humans than are rodents [154].
